# 

**Published:** 1877-04

**Authors:** 


### NEW YORK STATE DENTAL SOCIETY.

The Dental Society of the State of New York will hold its ninth annual meeting in the capitol at Albany, on Wednesday and Thursday, May 9th and 10th, 1877.

SUBJECTS AND ESSAYISTS.

Management of Proximal Surfaces. S. G. Perry, New York.

Dental Associations. A. M. Holmes, Morrisville.

Dental *vs.* Medical Practice. A. C. Hawes, New York.

Necrosis, its Cause and Cure. W. H. Atkinson, New York.

Artificial Dentures. J. G. Ambler, New York.

Dental Science. S. B. Palmer, Syracuse.

Necessity for Dental Legislation. C. P. Crandall, Brooklyn.

Climatic Influences upon the Teeth. G. C. Daboll, Buffalo.

Is it Correct Practice? W. C. Barrett, Buffalo.

---

### ILLINOIS STATE DENTAL SOCIETY.

The thirteenth annual meeting of the Illinois State Dental Society will be held in the "old supreme court room," Springfield, Illinois, commencing on Tuesday, May 8th, 1877, at ten o'clock a. m.

SUBJECTS FOR DISCUSSION.

1. The wants of the profession. K. B. Davis, Springfield.

2. Mechanical dentistry. C. B. Rohland, Alton.

3. Relative merits of certain materials for filling Teeth. J. F. Marriner, Ottawa.

4. Operative dentistry—Special points essential to success. J. N. Crouse, Chicago.

5. Mastication—A function to be valued, and a process to be taught. L. C. Ingersoll, Keokuk, Iowa.

6. Irregularities. Geo. H. Cushing, Chicago.

7. The tooth pulp and its treatment. E. D. Swain, Chicago.

8. Treatment of exposed pulps. T. W. Brophy, Chicago.

9. Treatment of first permanent molars, when decayed.

10. Literature. C. Stoddard Smith, Elgin.

11. Use and abuse of the mallet.

The executive committee would say that some new features have been introduced in the plans for this meeting which they think will add largely to its interest, and they are not without hope that this may prove the most interesting and profitable meeting this society ever held.

Hotel accommodations, Leland House, two and a half dollars per day, St. Nicholas, two dollars per day.

### MERRIMACK VALLEY DENTAL ASSOCIATION.

The fourteenth semi-annual meeting of the Merrimack Valley Dental Association will be held at Concert Hall, No.

100 Market street, Lynn, Mass., Thursday and Friday, May 3d and 4th, 1877, commencing at eleven a. m., on Thursday.

ESSAYISTS.

Dr. T. H. Chandler, of Boston, Mass.; Dr. D. B. Ingalls, of Clinton, Mass.; Dr. J. Guttman, of Great Falls, N. H.

CLINICAL OPERATORS.

Dr. I. A. Salmon, of Boston, Mass.; Dr. T. Haley, of Biddeford, Me.

SUBJECTS FOR DISCUSSION.

Dental irregularities, with a view to a clear and definite understanding of remedial methods.

Each member of the association is earnestly requested to prepare and present models with the correctional fixtures attached, representing one or more cases in actual practice. It is believed by the committee that such a comparison of methods will tend to evolve a more uniform and simple system in this important department of dental surgery.

The committee would also recommend that part of Thursday evening be devoted to exhibition of microscopes and microscopical subjects, and invite all members having either to bring them and report to Dr. R. R. Andrews, of Cambridge, Mass.

Hotel headquarters at Kirtland House, at reduced rates.

W. E. Riggs,
Secretary.

### EASTERN INDIANA DENTAL ASSOCIATION.

The sixth semi-annual meeting of the Eastern Indiana Dental Association will be held in Shelbyville, Tuesday and Wednesday, May 8th and 9th, 1877.

SUBJECTS FOR DISCUSSION.

1. Anatomy of the Oral Cavity and its Surroundings.
2. Abrasion of the Teeth—Chemical and Mechanical.
3. Mechanical Dentistry.

4. Microscopy.

5. Extraction of the First Permanent Molars: When Indicated.

6. Inflammation and Absorption of the Gums and Alveolus.

7. Filling Teeth: Comparative Value of Different Materials, and Different Methods of Operating.

Clinics will be held at such times during the meeting as the association may determine.

A full attendance is respectfully urged, and all members of the profession, whether members of the association or not, are cordially invited to be present, and will receive a hearty welcome.

### DENTAL SOCIETIES.

The eighth annual meeting of the California State Dental Society will be held in San Francisco, commencing Tuesday, June 5th, 1877, at ten o'clock a. m., and continue four days.

In addition to the regular reports of standing committees, papers will be presented on the following subjects, viz:

"What is to be the Future of Dentistry?" Dr. S. W. Dennis.

"Professional Etiquette." Dr. H. J. Plomteaux.

"The Diseases of the Gums and Processes, and the Treatment." Dr. S. E. Goe.

"The Best Methods and Materials for Treating and Filling Roots of the Teeth." Dr. S. E. Knowles.

"Treatment of Sensitive Dentine." Dr. C. L. Goddard.

All persons are respectfully invited to present voluntary essays, on any subject pertaining to the science or practice of the profession which they may select.

This society has a well deserved reputation for good and efficient work, and the indications are that the approaching meeting will equal, if not excel all previous ones.

A MONTHLY MAGAZINE DEVOTED TO THE INTERESTS OF

THE DENTAL PROFESSION.

*TERMS REDUCED TO $2.50 PER YEAR. SINGLE COPIES 25C.*

EDITED BY PROF. J. TAFT, D.D.S.

AND

*Published by SPENCER, CROCKER & CO., Ohio Dental Depot.*

The volume commences in January and closes in December.

The REGISTER being issued monthly is always fresh, therefore Indispensable to the practitioner who desires to keep posted up with the new inventions and improvements which are daily developed. Neither expense nor effort will be spared to give value and variety to its contents. Articles will be illustrated with well executed engravings whenever they will add to the interest or assist to explanation.

Its extensive circulation and frequency of issue make it one of the BEST DENTAL ADVERTISING MEDIUMS in the United States.

All subscriptions must begin with January or July.

Local or special notices, five lines or less, will be inserted for$3 each insertion; over five lines, 50 cts. per line.

All advertising bills payable in advance, or at furthest, after the issue of the first number containing the advertisement. All transient advertisements to be paid for in advance.

☞CONTRIBUTIONS SOLICITED.

Exclusively Editorial Communications should be addressed to Editor.

The latest number of the REGISTER may be ordered with or without other goods at any time, and all letters should be addressed to

***SPENCER, CROCKER & CO , Ohio Dental Depot, Cincinnati, O.***

